# Impact of rapid MALDI-TOF MS and susceptibilities on hospitalized children with Gram-negative bloodstream infections

**DOI:** 10.1128/spectrum.02922-25

**Published:** 2026-03-17

**Authors:** Maria E. Santos, Claire Justin, Margaret J. Trost, Jeffrey Bender, Choo Phei Wee, Kanokporn Mongkolrattanothai, Jennifer Dien Bard

**Affiliations:** 1 Division of Hospital Medicine, Department of Pediatrics, Children's Hospital Los Angeles 549365 , Los Angeles, California, USA; 2 Keck School of Medicine, University of Southern California 12223, Los Angeles, California, USA; 3 Kaiser Permanente 23543 , Los Angeles, California, USA; 4 Department of Pediatrics, City of Hope Comprehensive Cancer Center 20220 , Duarte, California, USA; 5 Southern California Clinical and Translational Science Institute, University of Southern California 5116, Los Angeles, California, USA; 6 Department of Pathology and Laboratory Medicine, Children's Hospital Los Angeles 635205, Los Angeles, California, USA; Indiana University School of Medicine, Indianapolis, Indiana, USA

**Keywords:** MALDI-TOF MS, pediatric, Gram-negative blood stream infections (GN-BSIs), rapid susceptibility testing, diagnostic testing

## Abstract

Matrix-assisted laser desorption ionization-time of flight mass spectrometry (MALDI-TOF MS) offers fast identification to improve time to appropriate antibiotics in Gram-negative bloodstream infections (GN-BSIs). This retrospective study evaluated patients with GN-BSI PRE- and POST-implementation of rapid identification with and without direct antimicrobial susceptibility testing (AST). Patients aged 0–18 years with GN-BSI before and after implementation of direct blood culture broth MALDI-TOF MS and AST were evaluated. Cohorts were defined by methods of organism identification and AST: traditional culture and AST methods (PRE), direct from broth identification by MALDI-TOF MS with culture-dependent AST (MALDI + cAST) and direct from broth identification MALDI-TOF MS with direct AST (MALDI + dAST). A total of 246 pediatric patients were included, 78 PRE, 92 MALDI + cAST, and 76 MALDI + dAST. Both POST-implementation groups had decreased median time from blood culture collection to identification by over 24 h (16.1 h MALDI + cAST and 15.5 h MALDI + dAST vs 42.9 h PRE; *P* < 0.0001). MALDI + dAST had decreased median time to susceptibilities (34.3 h MALDI + dAST vs 44.4 h PRE and 51.4 h MALDI + cAST; *P* < 0.0001) and decreased time to optimal therapy (TTOT) compared with PRE and MALDI + cAST (51.4 h MALDI + dAST vs 57.1 h PRE and 58.2 h MALDI + cAST; *P* = 0.03). There was no significant difference in time to effective therapy (TTET). Time to discontinuation of Gram-positive coverage decreased in MALDI + cAST and MALDI + dAST cohorts (39.3 h MALDI + cAST and 37.1 h MALDI + dAST vs 49.1 PRE; *P* = 0.03). The implementation of a rapid ID and AST approach significantly reduced the time to identification and time to susceptibility. However, a 17-h gap in antibiotic optimization highlights the need for additional interventions.

**IMPORTANCE:**

Rapid pathogen identification and susceptibility testing is essential for optimizing antimicrobial therapy in pediatric Gram-negative bloodstream infections. This study demonstrates that direct from blood culture broth identification with MALDI-TOF MS along with direct AST led to faster time to susceptibilities and time to optimal therapy in patients with GN-BSI. Despite this, there exists a 17-h gap between results and optimization of antibiotics. This suggests rapid diagnostic testing has not been effectively integrated into clinical practice and the need for active antimicrobial stewardship to improve the impact of these faster laboratory diagnostics.

Gram-negative bloodstream infections (GN-BSIs) are a leading cause of morbidity and mortality in hospitalized pediatric patients (1–3). Improving time to effective therapy (TTET) is imperative as delays of even 3 h have been associated with a fourfold increase in mortality (4–10). Current developments in medical technology have introduced novel rapid diagnostic testing such as matrix-assisted laser desorption/ionization time of flight mass spectrometry (MALDI-TOF MS), which allows for identification of bacteria and yeast directly from blood culture in a matter of hours (11–13). Literature in adult patients has found that implementing MALDI-TOF MS leads to earlier organism identification, improved TTET, decreased exposure to broad-spectrum antibiotics, and improved clinical outcomes (8, 14–18). Although limited, studies in children have also shown similar improved turnaround time (TAT) to organism identification and TTET with MALDI-TOF MS combined with an antimicrobial stewardship program (ASP) (12, 19–22). However, rapid testing for pathogen identification has outpaced that of antimicrobial susceptibility testing (AST), despite its critical impact on definitive therapy. Interpretation of genotypic resistance markers in Gram-negative pathogens has proven more challenging than in Gram-positive organisms (23). To our knowledge, there is sparse literature evaluating the impact of rapid AST combined with MALDI-TOF MS in pediatric patients with GN-BSI. We previously reported the impact of rapid AST on broad-spectrum antibiotic use and length of stay using a commercial panel in patients with GN-BSI; however, organisms tested by this method were limited (24).

The objective of the present study is to evaluate the impact of rapid identification and AST in pediatric patients hospitalized with GN-BSI. Expedited identification was performed directly from positive blood culture broth using MALDI-TOF MS and the impact of faster AST results was determined by comparing the difference between culture-dependent phenotypic AST and culture-independent AST (direct AST).

## MATERIALS AND METHODS

### Study design

This was a retrospective cohort study evaluating patients aged 0–18 years with GN-BSI PRE- and POST-implementation of MALDI-TOF MS over two consecutive time periods from December 2011–December 2013 (PRE) and January 2014–December 2016 (POST) at Children’s Hospital Los Angeles (CHLA). The PRE-implementation period consisted of standard of care testing which included culture-dependent workup for both identification and AST. The POST-implementation period consisted of two separate subgroups that included culture-dependent and culture-independent AST. These cohorts are further defined below.

### Data collection

Patients were identified through the electronic medical record (EMR) for the designated study period. The data collected included patient demographic information, such as age, sex, location, primary diagnosis, and comorbidities. Clinical information was collected, including hospital length of stay (LOS), ICU admission and LOS, 30-day mortality, BSI recurrence, central line removal, and Infectious Diseases (ID) consult. Microbiological information, including time to positive culture, identification, and AST, was also obtained. The presence of multidrug-resistant Gram-negative organisms, including extended-spectrum beta-lactamase (ESBL) producers, was also noted. Progress notes were reviewed to further evaluate for concurrent illness or other clinical factors that would influence antibiotic regimen. All primary and secondary outcome data were collected retrospectively.

### Exclusion criteria

Only blood cultures positive for Gram-negative organisms were included in the study. Patients were excluded if they had polymicrobial BSI, if the positive blood culture was considered a contaminant, or if the patient was discharged prior to positive result. Episodes of bacteremia were excluded if the patient was already being treated for bacteremia. If the patient was being treated for a concomitant infection, they were also excluded. If multiple blood cultures were drawn simultaneously for one episode of BSI, then the sample with the fastest TAT was used to evaluate outcome variables. Unique episodes of BSI from the same patient were included if they met the above criteria.

### Cohort definitions

**Fig 1:**
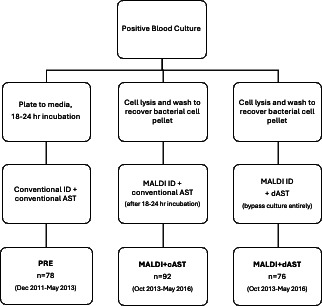
Identification and antibiotic susceptibility testing approaches for the three cohorts from blood culture positive results. PRE, MALDI + cAST, MALDI + dAST. Summary of the three separate protocols pursued. The PRE group consisted of standard of care, culture-independent methods after 18–24 h subculture. The MALDI + cAST group consisted of direct from blood culture broth identification by MALDI-TOF MS and culture-dependent antimicrobial susceptibility testing after 18–24 h subculture. The MALDI + dAST group consisted of direct from blood culture broth identification and AST. This bypassed 18–24 h subculture.

The patients were divided into two time periods: (i) PRE, which utilized conventional culture-dependent methods for both organism identification and AST, and (ii) POST, which utilized MALDI-TOF MS directly from positive blood culture for organism identification. The POST group was further separated into two subgroups: (a) MALDI + cAST, which utilized MALDI-TOF MS directly from positive blood culture for organism identification and conventional culture-dependent methods for AST (cAST), and (b) MALDI + dAST, which utilized MALDI-TOF MS directly from positive blood culture for organism identification followed by culture-independent direct AST (dAST) (Fig. 1).

### Microbiology workup

All blood cultures were obtained and incubated in the BacT/ALERT (bioMérieux, Durham, NC) or BD Bactec FX system (BD, Franklin Lakes, NJ) automated blood culture system. Blood collection practices remained consistent throughout the three cohort periods. Upon blood culture positivity, Gram stains are performed, called to the treating physician, and reported in the EMR within 1 h.

In the PRE period, positive blood cultures were subcultured onto solid agar, and organism identification was carried out using conventional methods, including Vitek II (bioMérieux) and other biochemical tests. Routine AST was performed using Vitek II, Etest (bioMérieux) and/or Sensititre broth microdilution (Thermo Fisher Scientific, Waltham, MA). Screening for ESBL was performed per CLSI protocol (25). Final identification and susceptibility results were reported in EMR.

In the POST period, MALDI-TOF MS was performed using a lysis-centrifugation step directly from positive blood culture broth that was previously developed (26). This is split up into two separate subgroups, as follows. (a) MALDI + cAST: identification performed directly from blood culture broth but AST still required incubation of media for 18–24 h prior to setting up. The AST methods used included Phoenix (BD), Etest (bioMérieux), and/or Sensititre broth microdilution (Thermo Fisher). (b) MALDI + dAST: utilized the same lysis centrifugation step to generate a cell pellet for both organism identification and culture-independent AST approach. Briefly, a 0.5 McFarland was made from the MALDI bacterial cell pellet, and AST was performed using the Phoenix system (BD). This bypassed the need to incubate blood culture broth for 18–24 h prior to setting up AST.

Both direct MALDI TOF-MS and direct AST were offered 24/7 but were subject to clinical workload and staffing availability, sufficient cell pellet for AST, and MALDI-TOF MS score of ≥1.7 to allow for high confidence identification to at least the genus level. Susceptibility results from Vitek II may be available as early as 5 h from start time compared with the standard 16–18 h for the other AST approaches including Phoenix.

### Antimicrobial stewardship program

During the study period, the ASP underwent expansion from an initial focus on oncology units prior to 2014 to a hospital-wide service with prospective audit and feedback to date. In addition, blood culture notification via email was initiated in 2014 to notify the team of any new blood culture positives. This was in addition to the critical call notification of the initial Gram stain result to the physician. Positive blood culture results were reviewed by an ASP physician Monday to Friday during working hours, and ASP contacted the treating physician for antibiotic recommendation when appropriate. AST results were reported in EMR only. Formal ID consultation was not mandated but rather recommended by ASP in patients with GN-BSI with severe illness requiring ICU admission, immunocompromise, or a complicated clinical course.

### Primary outcomes

#### Time to effective therapy

TTET was measured from the time of blood culture collection to the first dose of an antimicrobial with known susceptibility results. Therapy was considered effective when the organism was susceptible to the antibiotic based on microbiology report, site of infection, drug route, and drug dosing.

#### Time to optimal therapy

Calculation of time to optimal therapy (TTOT) was measured for the gram-negative bacteremia from the time of blood culture collection to the first dose of the narrowest therapy for given infection based on identified pathogen, AST, and site of infection. Optimal therapy can be defined by (i) transitioning from a broad spectrum to a narrower spectrum agent and vice versa, (ii) deescalating antibiotics to a single agent, and (iii) tailoring dosing and route to align with institution guidelines. If a formal ID consult was made during the episode of bacteremia, appropriate therapy was based on their formal recommendations in the EMR. For complicated cases without ID consult, pediatric hospitalist (MS) and ID physician (KM) would decide on effective and optimal antimicrobial regimen on a case-by-case basis. If after 7 days a patient was not on optimal treatment, their results were recorded as “never on optimal therapy.”

#### Time to de-escalation of Gram-positive coverage

Unnecessary Gram-positive coverage was defined as empiric therapy with either vancomycin or linezolid after GN-BSI was detected. Time to de-escalation of Gram-positive coverage was defined as time to first dose of vancomycin or linezolid to the time of order discontinuation.

### Secondary outcomes

Secondary outcomes included: time to identification and AST (from time of blood culture collection), hospital LOS, ICU admission and LOS, ID consult, recurrence of bacteremia, central line removal, 30-day mortality, and presence of multidrug-resistant organism (MDRO) or ESBL. ICU admission was defined as any admission within 24 h of blood culture or during treatment of BSI. Recurrence was classified as any positive within 30 days after completing treatment of previous BSI for the same organism. Central line removal was defined as any removal occurring within the first 48 hours following blood culture positivity. MDROs were defined as any organism that was resistant to three or more antimicrobial classes, one of which must include a third-generation cephalosporin.

### Statistical analysis

This study had three primary analytic endpoints and five secondary analytic endpoints as referenced above. Summary statistics, such as median with interquartile range and frequency and percentage, were used to describe the distribution of study variables. Demographic, comorbidity, and clinical variables were compared among PRE, MALDI + cAST and MALDI + dAST and analyzed with Chi-Squared test and Fisher’s Exact test when the expected number of observations of one of the cells was less than five in contingency table analysis for categorical data. The Kruskal-Wallis test was used for statistical comparison for skewed continuous data. A subgroup analysis of demographics and clinical variables of patients with MDRO GN-BSI was analyzed with Chi-Squared test, Fisher’s Exact test when the expected number of observations of one of the cells was less than five in contingency table analysis for categorical data. Wilcoxon Rank-Sum test was used for statistical comparison for skewed continuous data. Then, Kaplan-Meier analysis with Log-rank test was used for time to event comparisons among PRE, MALDI + cAST, and MALDI + dAST. Multivariate Cox regression analysis was used to assess the influence of MALDI-TOF on TTOT after controlling for clinical characteristics. All tests were two-sided with significance level set at *P* < 0.05. Statistical computations were performed using Stata/SE 15.1 (StataCorp, College Station, TX).

## RESULTS

### Demographics

Our study included 246 BSI from 242 unique patients divided into three cohorts: 78 PRE, 92 MALDI + cAST, and 76 MALDI + dAST. Baseline demographics, such as age, sex, comorbidities, presence of central line, and location, were similar except the MALDI + cAST group was younger than MALDI + dAST and PRE (2.5 years MALDI + cAST vs 6 years MALDI + dAST and 8 years PRE; *P* < 0.001, Table 1). Most patients were immunocompromised (53%) and had a central line (80%). *Escherichia coli* was the most common pathogen overall (81, 33%) and the MDRO rate across cohorts ranged from 5% to 15%.

**TABLE 1:** Demographic and clinical characteristics of pediatric patients with GN-BSI^*a*^

	Total *n* (%)	PRE (*n* = 78) *n* (%)	MALDI + cAST (*n* = 92) *n* (%)	MALDI + dAST (*n* = 76) *n* (%)	*P*
Age (in years), median (IQR)	5, 1–14	8, 1–16	2.5, 0.71–8	6, 2–14.5	0.001^*d*^
Gender					
Male	141 (57.3)	44 (56.4)	55 (59.8)	42 (55.3)	0.825^*c*^
Female	105 (42.7)	34 (43.6)	37 (40.2)	34 (44.7)	
Co-morbidities					0.614^*b*^
None	43 (17.5)	14 (18.0)	20 (21.7)	9 (11.8)	
Immunosupp (non onc)	20 (8.1)	8 (10.3)	4 (4.3)	8 (10.5)	
Immunosupp (onc)	110 (44.7)	33 (42.3)	43 (46.7)	34 (44.7)	
TPN	42 (17.1)	12 (15.4)	15 (16.3)	15 (19.7)	
Cardiac	19 (7.7)	5 (6.4)	8 (8.7)	6 (7.9)	
Heme	11 (4.5)	5 (6.4)	2 (2.2)	4 (5.3)	
Dialysis dependent	1 (0.4)	1 (1.3)	0 (0.0)	0 (0.0)	
Location					0.083^*b*^
General pediatric floor	100 (40.7)	28 (35.9)	41 (44.6)	31 (40.8)	
Onc/BMT	125 (50.8)	48 (61.5)	41 (44.6)	36 (47.4)	
ICU	21 (8.5)	2 (2.6)	10 (10.9)	9 (11.8)	
Presence of central line					0.085^*b*^
No	50 (20.3)	20 (25.6)	16 (17.4)	14 (18.4)	
Central venous catheter	157 (63.8)	50 (64.1)	56 (60.9)	51 (67.1)	
Peripherally inserted catheter	33 (13.4)	8 (10.3)	14 (15.2)	11 (14.5)	
Hemodialysis catheter	6 (2.4)	0 (0.0)	6 (6.5)	0 (0.0)	
Central line removed					0.012^*c*^
No	145 (58.9)	51 (65.4)	53 (57.6)	41 (54.0)	
Yes	52 (21.1)	7 (9.0)	24 (26.1)	21 (27.6)	
Not applicable	49 (19.9)	20 (25.6)	15 (16.3)	14 (18.4)	
Infectious disease consult					<0.0001^*c*^
No	145 (58.9)	63 (80.8)	46 (50.0)	36 (47.4)	
Yes	101 (41.1)	15 (19.2)	46 (50.0)	40 (52.6)	
Recurrence of bacteremia					0.363^*b*^
No	235 (95.5)	74 (94.9)	90 (97.8)	71 (93.4)	
Yes	11 (4.5)	4 (5.1)	2 (2.2)	5 (6.6)	
Length of stay, median (IQR)	16.5 (9–46)	17 (10–42)	17 (8.5–56.5)	16 (9–33.5)	0.678^*d*^
Peds ICU admission					0.100^*b*^
No	178 (72.4)	64 (82.1)	65 (70.7)	49 (64.5)	
Yes	64 (26.0)	13 (16.7)	25 (27.2)	26 (34.2)	
CTICU or NICU	4 (1.6)	1 (1.3)	2 (2.2)	1 (1.3)	
PICU length of stay, median (IQR)	4 (3–13)	4.5 (3–10)	4 (3–16)	6 (3–13)	0.982^*d*^
Mortality within 30 days of positive blood culture					0.113^*b*^
No	233 (94.7)	77 (98.7)	86 (93.5)	70 (92.1)	
Yes	13 (5.3)	1 (1.3)	6 (6.5)	6 (7.9)	
On effective empiric therapy					0.073^*c*^
No	37 (15.0)	8 (10.3)	20 (21.7)	9 (11.8)	
Yes	209 (85.0)	70 (89.7)	72 (78.3)	67 (88.2)	
Multidrug-resistant organism					0.145^*c*^
No	220 (89.4)	74 (94.9)	81 (88.0)	65 (85.5)	
Yes	26 (10.6)	4 (5.13)	11 (12.0)	11 (14.5)	
Extended spectrum beta-lactamase producer					0.566^*b*^
No	231 (93.9)	75 (96.2)	85 (92.4)	71 (93.4)	
Yes	15 (6.1)	3 (3.9)	7 (7.6)	5 (6.6)	
Organism identification					0.228^*b*^
*Escherichia coli*	81 (32.9)	32 (41.0)	23 (25.0)	26 (34.2)	
*Enterobacter cloacae*	30 (12.2)	6 (7.7)	16 (17.4)	8 (10.5)	
*Klebsiella pneumoniae*	56 (22.8)	17 (21.8)	24 (26.1)	15 (19.7)	
*Pseudomonas aeruginosa*	32 (13.0)	6 (7.7)	14 (15.2)	12 (15.8)	
*Serratia marcescens*	9 (3.7)	5 (6.4)	3 (3.3)	1 (1.3)	
*Klebsiella oxytoca*	13 (5.3)	5 (6.4)	5 (5.4)	3 (4.0)	
Other	25 (10.2)	7 (9.0)	7 (7.6)	11 (14.5)	

^*a*^ GN-BSI, Gram-negative bloodstream infection.

^*b*^ Fisher’s exact test is used for statistical comparison.

^*c*^ Chi-squared test is used for statistical comparison.

^*d*^ Kruswal-Wallis test is used for statistical comparison among PRE, MALDI, and MALDI + dAST.

Implementation of MALDI-TOF MS significantly decreased the time from blood culture collection to organism identification in both MALDI + cAST and MALDI + dAST (16.1 h MALDI + cAST and 15.5 h MALDI + dAST vs 42.9 h PRE; *P* < 0.0001, Table 2). When comparing time from blood culture positivity to organism identification, the median time to identification was 2.0 h for both MALDI + cAST and MALDI + dAST (2 h MALDI + cAST and 2 h MALDI + dAST vs 28 h in PRE, *P* < 0.0001, Table 2). Time from blood culture collection to AST reporting was faster in MALDI + dAST compared with PRE and MALDI + cAST (34.3 h MALDI + dAST vs 44.4 h PRE vs 51.4 h MALDI + cAST) (*P* < 0.0001).

**TABLE 2:** Differences in children with GN-BSI pre/post implementation of MALDI-TOF MS^*a*^^,^^*b*^

	PRE *n* = 78	MALDI + cAST *n* = 92	MALDI + dAST *n* = 76	*P*-value
Time from BC collection to identification	42.9 (37.8–48.3)	16.1 (12.2–18.7)	15.5 (12.3–19.6)	<0.0001
Time from BC positivity to identification	28.0 (23.3–34.0)	2.0 (1.0–3.0)	2.0 (1.0–3.0)	<0.0001
Time from BC collection to susceptibilities	44.4 (39.4–54.9)	51.4 (45.5–58.1)	34.3 (29.5–37.5)	<0.0001
Time to optimal therapy^*c*^	57.1 (44.2–75.2)	58.2 (45.2–79.6)	51.4 (38.9–62.1)	0.03
Time to effective therapy	0.12 (0.02–2.78)	0.02 (0.02–4.58)	0.03 (0.02–4.27)	0.93
Discontinuation of vancomycin^*d*^	49.1 (34.3–55.5)	39.3 (22.5–50.5)	37.1 (21.7–50.8)	0.05

^*a*^ GN-BSI, Gram-negative bloodstream infection; MALDI-TOF MS, matrix-assisted laser desorption/ionization time-of-flight mass spectrometry; BC, blood cultures.

^*b*^ All values in hours, median (interquartile range).

^*c*^ Total N varied: PRE = 69, MALDI = 92, MALDI + dAST = 75.

^*d*^ Total N varied: PRE = 46, MALDI = 61, MALDI + dAST = 48.

### Time to effective therapy

Overall, 209/246 of patients were on effective empiric antibiotic therapy at the time of blood culture collection. There was a slight improvement in TTET among MALDI + cAST and MALDI + dAST when compared with PRE, though not statistically significant. When looking at the 37 patients not on effective empiric antibiotics, 20 in the MALDI + cAST group had significantly faster time to effective therapy than the eight patients in the PRE group. The remaining nine patients in MALDI + dAST experienced longer TTET (18.1 h PRE vs 11.35 h MALDI + cAST vs 21.1 h MALDI + dAST; *P* < 0.0001). This subgroup included mostly patients who had cardiac disease (10), were immunosuppressed (13), and had a central line (33). Seven ultimately died, and almost a third had MDRO-BSI.

### Time to optimal therapy

The MALDI + dAST group had the shortest TTOT among the three groups, by a difference of 6 median hours compared with the PRE group . In contrast, the TTOT between MALDI + cAST and PRE was comparable (51.4 h MALDI + dAST vs 58.2 h MALDI + cAST vs 57.1 h PRE). Ten patients were excluded because they were never on optimal therapy; nine were in the PRE cohort, and two ultimately died. In a multivariate Cox regression model looking at TTOT and controlling for age, gender, location, and ID consult, the difference remained significant (HR = 1.61, CI = 1.12–2.33, *P* = 0.011, Table 3). When looking at a subgroup of 37 patients not on effective empiric antibiotics, MALDI+AST resulted in a ~16-h decrease in median time to optimal therapy (39.4 h, 95% CI, 17.6 to 97.3) when compared with the PRE (55.8 h, 95% CI, 37.3 to 60.1) and MALDI cohorts (56.5 h, 95% CI, 42.1 to 76.9). Overall, MALDI + dAST did not impact TTET; however, it did improve TTOT, particularly in patients not initially on effective therapy.

**TABLE 3:** Multivariate Cox regression model for time to optimal therapy^*a*^

	Hazard ratio	95% CI	*P*
MALDI TOF			
PRE (Ref)	1.00		
DM	1.26	(0.88–1.78)	0.203
DMS	1.61	(1.12–2.33)	0.011
Age^*b*^	1.01	(0.98–1.03)	0.608
Gender			
Male (Ref)	1.00		
Female	0.96	(0.73–1.26)	0.747
Location			
Non oncology/BMT unit (Ref)	1.00		
Oncology/BMT unit	0.84	(0.63–1.13)	0.247
Identification consult			
No (Ref)	1.00		
Yes	0.95	(0.71–1.27)	0.720

^*a*^ (Ref) denotes comparison group.

^*b*^ Age is treated and analyzed as continuous.

### Time to de-escalation of Gram-positive coverage

To evaluate the impact on de-escalation of Gram-positive coverage for GN-BSI, time on vancomycin or linezolid was measured. Empiric Gram-positive coverage was initiated on 155 patients (PRE=46, MALDI + cAST = 61, MALDI + dAST = 48). When compared with the PRE cohort, the median time to discontinuation of Gram-positive coverage decreased (from 49.1 h PRE to 39.3 h MALDI + cAST and 37.1 h MALDI + dAST; *P* = 0.05) in the MALDI and MALDI + dAST cohorts.

### Secondary outcomes

Clinical factors, such as hospital LOS, PICU admission and LOS, 30-day mortality, 30-day recurrence, central line removal, ID consult, and effective initial empiric therapy, were collected. Notable clinical factors for PRE, MALDI + cAST and MALDI + dAST, respectively, included less central line removals (9% vs 26.1% vs 27.6%; *P* = 0.012) and less ID s (19.2% vs 50% vs 52.6%; *P* < 0.0001) in the PRE period (Table 1). Overall, the recurrence rate was 4.5%, the mortality rate was 5.3%, and 85% of patients were ordered for effective empiric therapy at the time of blood culture collection. Remaining clinical factors were not significant.

## DISCUSSION

This study demonstrated that implementation of culture-independent phenotypic susceptibility testing (MALDI + dAST) led to shorter time to identification, AST reporting, and optimal therapy in patients with GN-BSI. Additionally, both POST MALDI-TOF MS cohorts showed a 12-h decrease in time to discontinuation of unnecessary Gram-positive coverage. While other pediatric studies have demonstrated up to an hour decrease in TTET, our study revealed that all patients at our institution received early broad-spectrum antibiotics, aligning with institutional empiric guidelines (20, 21). However, for patients that were not on effective empiric antibiotic therapy, the use of rapid AST improved TTOT by almost 16 h, while TTET was only improved in the MALDI + cAST group. Our rates for ID consults were higher in the POST-MALDI-TOF MS period (both MALDI + cAST and MALDI + dAST groups), potentially indicating a change in culture in the age of novel diagnostic testing, increasing rates of MDRO, and a more robust ASP. Despite this, the effect of the MALDI-TOF MS remains evident when controlling for ID consults.

While AST results reported at a median of 34 h, optimal antibiotics were not initiated until a median of 51 h post-blood culture draw, indicating a 17-h delay in action. Moreover, there was a 20-h gap between Gram-negative organism identification and Gram-positive antimicrobial de-escalation, suggesting that rapid diagnostic testing has not been effectively integrated into our pediatric clinical practice. Similarly, in a study evaluating the impact of rapid AST on antimicrobial use, they reported opportunities to narrow therapy were less utilized than opportunities to broaden therapy (22). This could result from physician hesitancy to tailor antibiotic therapy based on rapid diagnostic testing alone or reflect a desire to rule out potential polymicrobial infections. It is possible that if ASP had been available 24/7, the impact of MALDI-TOF MS could have been more significant as real-time feedback could have swayed physicians to tailor antibiotics sooner.

It has been well established that implementation of rapid diagnostic testing alongside ASP is superior to rapid diagnostic testing alone (8, 10, 27, 28). Previous studies evaluating the impact of MALDI-TOF MS with ASP in adults demonstrate reduction of TTET and TTOT, decreased hospital LOS, ICU LOS, recurrent bacteremia, and all-cause mortality (8, 18, 27, 29). Pediatric studies revealed similar trends in TTOT yet failed to show a statistically significant change in patient outcomes (12, 19–21). In two pediatric studies, an 8–20-h delay between initiating optimal therapy and diagnostic results in the presence of ASP was reported, suggesting areas for improvement not currently addressed by ASP (19, 20). In our study, consistent ASP in the MALDI + cAST and MALDI + dAST cohorts appears to be inadequate to completely address the discrepancy between test results and clinician action. Further prescriber education, ID consultation, optimization of EMR, and quality improvement initiatives have been proposed as an adjunct to ASP efforts to help bridge this gap (30–32).

One unique outcome measure of our study was the discontinuation of vancomycin in GN-BSI. While Reuter et al. report approximately 13-h decrease in vancomycin for patients with methicillin-sensitive *Staphylococcus aureus*, our study focuses on the use of inappropriate Gram-positive coverage in patients with GN-BSI (19). One study from 2003 found that vancomycin was unnecessarily initiated in approximately 50% of pediatric inpatients with a median duration of 72 h (33). With rising rates of vancomycin-resistant organisms, stewardship efforts call for more judicious use of vancomycin therapy (34, 35). Our study demonstrated that in the post-intervention groups, patients received approximately two fewer doses of vancomycin and had an average duration of empiric therapy of 37–39 h overall. While not addressed in this study, future studies can further evaluate the impact of reducing unnecessary Gram-positive coverage on patients’ risk of acute kidney injury and rates of vancomycin resistance.

Our study has several limitations. Our study population may not be extrapolated to other centers given the complexity of patients and high rates of resistance. Due to the limitations of retrospective review, despite controlling for patients with similar demographics, we are unable to adjust for all possible confounders or changes in clinical practices between pre- and post-intervention periods. For instance, ASP was more structured during the MALDI + cAST and MALDI + dAST cohorts compared with the PRE cohort, which may account for some of the improvements seen. However, TTOT was still significantly improved in the MALDI + dAST compared with the MALDI + cAST group, arguing that the shortened time to susceptibility was secondary to our intervention. While there was a statistically significant increase in ID consults in our post-intervention period, we controlled for this variable in our multivariate statistical analysis. Furthermore, there were uncontrolled variables, such as clinical workload and staffing, that may have prevented the laboratory staff from performing MALDI and/or AST directly from blood culture broth, which could have resulted in selection bias. Additionally, cell pellets that are insufficient to generate 0.5 McFarland solution would not proceed with dAST. The blood culture system was changed from BacT/ALERT to BD Bactec FX within the study time period; however, as we are only assessing the workup of patients with positive blood cultures, there should be no impact of bias expected. Further, there were no notable differences in median time to positivity between the two systems (BacT/ALERT, 13.9 h vs BD Bactec FX, 13.7 h, *P* = 0.411). Alongside the above change, the primary AST panel in the microbiology laboratory also changed from Vitek II in the PRE cohort to Phoenix in the remaining two cohorts, which requires an additional 10+ hours for results. The longer time to susceptibility results in the MALDI + cAST group compared with the PRE group is likely attributed to this change in system, and we would expect an even more dramatic improvement in the MALDI + dAST group had the systems not changed. Finally, MALDI-TOF MS identification and direct AST using cell pellet generated from blood culture broth is not a method that is approved by the Food and Drug Administration, and off-label validation is required prior to implementation in the clinical laboratory.

### Conclusion

The evolution of diagnostic testing has changed the landscape of clinical medicine and the successful integration of new technologies in existing practice is paramount. Despite faster time to identification and susceptibility results, there remains a 17–20-h delay in antibiotic optimization. This implies that diagnostic testing along with the aid of ASP cannot fully change clinical practice. This highlights an opportunity for alternative measures, such as prescriber education, introduction of EMR-based alerts, and quality improvement initiatives to bridge the gap between novel diagnostic testing and physician practice.
